# Serum activin A levels and tubal ectopic pregnancy

**Published:** 2014-03

**Authors:** Julio Elito Júnior, Leandro Gustavo Oliveira, Marcelo Octávio Fernandes Silva, Edward Araujo Júnior, Luiz Camano

**Affiliations:** *Department of Obstetrics, Federal University of São Paulo (UNIFESP), São Paulo-SP, Brazil.*

**Dear Editor**

Ectopic pregnancy (EP) is the most important cause of maternal death in the first trimester accounting for 9 - 13% of all pregnancy-related deaths (1). Despite the use of transvaginal sonography and high sensitive quantitative beta-hCG assays for diagnosis, nearly 40-50% of all EPs may be initially misdiagnosed (2). Therefore, several biomarkers have been investigated for early diagnosis of EP. Activin A, a dimeric glycoprotein belonging to the TGF- superfamily, has been highlighted among these “new biomarkers” (3). Florio *et al* reported that serum activin A levels can be markedly lower in women with tubal EP than those levels found either during normal intrauterine pregnancy or spontaneous miscarriage (4). The authors showed that a cut-off value of 0.37 ng/mL have 100% and 99.6% of sensitivity and specificity, respectively for predicting EP. Conversely, Kirk *et al* demonstrated in another elegant study that activin A has much lower sensitivity and specificity when used as a single biomarker for the diagnosis of EP (5). Bearing on these considerations, it is clear that this subject still needs to be carefully evaluated and it prompted us to develop this project where we compared serum activin A levels in women at first trimester of normal pregnancy to women with tubal EP.

**Figure 1:**
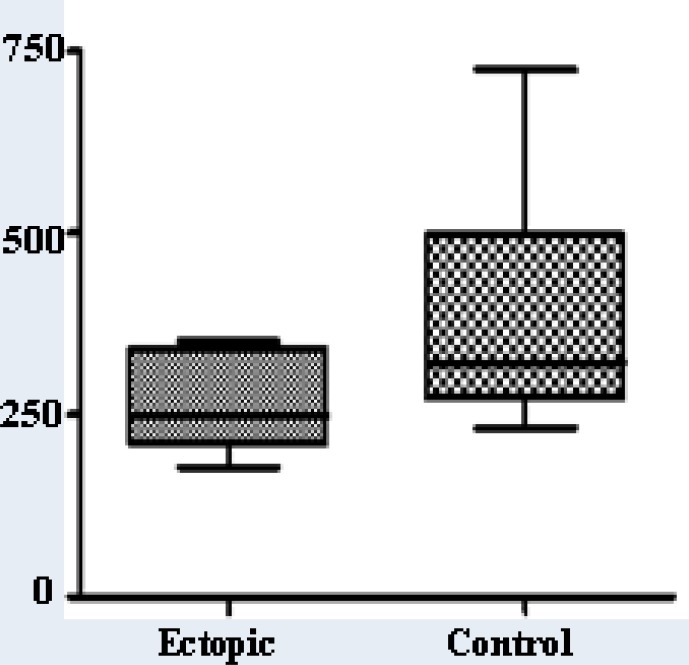
Serum levels of activin A in women with ectopic pregnancy and normal pregnancy at 5-6 weeks gestational age (pg/mL).

The study group was comprised of 5 women with EP. The diagnosis criteria was based on visualization of an adnexial mass by transvaginal scan associated with positive test for β-hCG in women with a suspected EP (amenorrhea, bleeding and pain). For this study were included only those cases in which the gestational age was established between 5-6 weeks gestational age. In all patients the mass was confirmed to be a tubal EP without signs of rupture by pathologist analysis. The control group was comprised of 10 women with normal intrauterine pregnancy at 5-6 weeks gestational age confirmed by transvaginal scan (evaluated by gestational sac measurements and/or crown-rump length). This work has been approved by the Ethics Committee of The Federal University of São Paulo (UNIFESP). Informed consent was obtained from all participants before the blood samples were collected. All blood samples of patients with EP were collected before surgery by peripheral venous puncture and immediately centrifuged at 1000g for 10 minutes; the supernants were stored at -80^o^C until processing. For the determination of serum activin A levels a commercially available assay for ELISA was used (activin A- Catalog number: Dy338; R&D systems, Inc. Minneapolis, USA). All activin A measurements and analysis were performed in duplicate at the same time. The limit of detection of the kit was less than 0.133 ng/mL. Data were presented as mean and standard deviation. The statistical analysis was performed using Prism software (version 4.02, Graph Pad Software Inc., San Diego, CA). The two groups were compared using Mann-Whitney U test and results were considered significant when p<0.05. The mean gestational age was 5.2±0.14 for the EP group and 5.2±0.19 for the normal intrauterine pregnancy group. The mean of activin A serum levels was 0.26±0.714 ng/mL and 0.37±161.4 ng/mL for women with EP and for women with normal pregnancy, respectively (p=0.206). The Figure 1 depicts all cases studied. 

Activin A has been reported to play an important role in the implantation process and inadequate levels of this molecule may be related to either an inappropriate implantation site or trophoblastic alterations (6). Therefore, some authors have investigated activin A as a biomarker for early diagnosis of EP. In this work we demonstrated that serum activin A levels could not discriminate between an EP from a normal intrauterine pregnancy (p=0.206) when an adnexial mass was found by transvaginal scan. These results are in agreement with those reported by Kirk *et al *regarding pregnancy of unknown location, although we had a different methodology (5).

Florio *et al* demonstrated that an activin A cut-off value of 0.37 ng/mL has 100% and 99.6% of sensitivity and specificity for diagnosis of EP (4). Despite we had a small number of cases it is possible to suggest that serum activin A may have a peculiar curve in EP as none of our results were higher than the cut-off value established by Florio *et al* even with identifiable adnexial mass (4). Therefore, we suggest that a multicentre study should be developed to assemble a good number of cases to properly define an activin A curve during EP. In such work other molecules (to be discussed) could be also investigated and we could finally discriminate whether some of them can really be used for early diagnosis of ectopic pregnancy. Meanwhile, we are continuing our investigation to better understand the relationship between activin A and EP.
